# A Simple Defined Medium for the Production of True Diketopiperazines in *Xylella fastidiosa* and Their Identification by Ultra-Fast Liquid Chromatography-Electrospray Ionization Ion Trap Mass Spectrometry

**DOI:** 10.3390/molecules22060985

**Published:** 2017-06-13

**Authors:** Michelli Massaroli da Silva, Moacir dos Santos Andrade, Anelize Bauermeister, Marcus Vinícius Merfa, Moacir Rossi Forim, João Batista Fernandes, Paulo Cezar Vieira, Maria Fátima das Graças Fernandes da Silva, Norberto Peporine Lopes, Marcos Antônio Machado, Alessandra Alves de Souza

**Affiliations:** 1 Departamento de Química, Universidade Federal de São Carlos, CP 676, 13565-905 São Carlos-SP, Brazil; mimassaroli@gmail.com (M.M.d.S.); msandrade2003@gmail.com (M.d.S.A.); mrforim@yahoo.com.br (M.R.F.); djbf@ufscar.br (J.B.F.); paulocezarv@gmail.com (P.C.V.); 2 Núcleo Pesquisas em Produtos Naturais e Sintéticos, Faculdade de Ciências Farmacêuticas de Ribeirão Preto, Universidade de São Paulo, 14040-903 Ribeirão Preto-SP, Brazil; ane_qui@hotmail.com (A.B.); npelopes@fcfrp.usp.br (N.P.L.); 3 Centro APTA Citros Sylvio Moreira, Instituto Agronômico, CP 04, 13490-970 Cordeirópolis-SP, Brazil; marcussilva727@uol.com.br (M.V.M.); marcos@centrodecitricultura.br (M.A.M.); alessandra@centrodecitricultura.br (A.A.d.S.)

**Keywords:** *Xylella fastidiosa*, *X. fastidiosa* medium XFM, diketopiperazines, Ultra-Fast Liquid Chromatograph, mass spectrometry, electrospray ionization

## Abstract

Diketopiperazines can be generated by non-enzymatic cyclization of linear dipeptides at extreme temperature or pH, and the complex medium used to culture bacteria and fungi including phytone peptone and trypticase peptone, can also produce cyclic peptides by heat sterilization. As a result, it is not always clear if many diketopiperazines reported in the literature are artifacts formed by the different complex media used in microorganism growth. An ideal method for analysis of these compounds should identify whether they are either synthesized de novo from the products of primary metabolism and deliver true diketopiperazines. A simple defined medium (*X. fastidiosa* medium or XFM) containing a single carbon source and no preformed amino acids has emerged as a method with a particularly high potential for the grown of *X. fastidiosa* and to produce genuine natural products. In this work, we identified a range of diketopiperazines from *X. fastidiosa* 9a5c growth in XFM, using Ultra-Fast Liquid Chromatography coupled with mass spectrometry. Diketopiperazines are reported for the first time from *X. fastidiosa*, which is responsible for citrus variegated chlorosis. We also report here fatty acids from *X. fastidiosa*, which were not biologically active as diffusible signals, and the role of diketopiperazines in signal transduction still remains unknown.

## 1. Introduction

*Xylella fastidiosa* has been associated with diseases of economically important crops including citrus, grapevine, plum, almond, peach, and coffee [1]. In *Citrus*, it is responsible for citrus variegated chlorosis (CVC), a disease that has caused millions of dollars of damage to the Brazilian citrus industry. The economic importance of the citrus industry in Brazil and the high level of damage caused by CVC in Brazilian orchards have resulted in an extensive research program starting with the sequencing of the entire genome of *X. fastidiosa* [2]. This Gram-negative bacterium is a fastidious organism that is able to colonize the xylem vessel of several host plants and the cibarium of sharpshooter leafhopper vectors [1].

Several *X. fastidiosa* genes show high levels of similarity to diverse *Xanthomonas campestris pv. campestris* genes, including genes associated with the synthesis and perception of a signal molecule that regulates the expression of pathogenicity factors such as plant cell wall degrading enzymes and extracellular polysaccharides [2]. It seems that a minimum *X. fastidiosa* cellular density in the host xylem needs to be reached so that CVC symptoms are observed. This fact strongly suggests that pathogenicity factor synthesis is dependent on a quorum-sensing mechanism.

The term quorum-sensing is used to describe a gene expression regulation mechanism according to cell population density fluctuation. Bacteria which present this mechanism produce and release signaling molecules called autoinducers or diffusible signal factors (DSF), which increase their concentration according to population density. In general, DSF may be chemically classified according to Gram’s stain. Thus, Gram-positive bacteria generally use peptides or amino acids as signaling molecules while Gram-negative ones usually produce fatty acid derivatives, such as *N*-acyl homoserine lactones derivatives [3].

*Xanthomonas campestris pv. campestris*, the causal agent of black rot disease, presents a quorum-sensing mechanism and the DSF synthesis is regulated by the regulation of pathogenicity factor (*rpf*) gene cluster. Production of xanthan gum, extra cellular enzymes, and cyclic glucones are regulated by *X. campestris pv. campestris* DSF and contribute to disease virulence. Its diffusible signal factor chemical structure was identified as *cis*-11-methyl-2-dodecenoic acid [4]. It has been shown that *X. fastidiosa* synthesizes a putative diffusible signal factor that activates regulation of pathogenicity factor (*rpf*) genes as in *X. campestris pv. campestris*. An investigation of *X. fastidiosa* strain 9a5c diffusible signal factor led to the identification of 12-methyltetradecanoic acid (by high-resolution gas chromatography-mass spectrometry, GC-MS). However, the authors worked only with acellular culture supernatant [5]. Beaulieu and collaborators [6] previously isolated 2-*cis*-tetradecenoic acid from a grape strain of *X. fastidiosa*, which was biologically active in the *Xf*DSF-biosensor strain [7]. However, the authors commented that in a bioassay with 12-methyltetradecanoic acid above (from *X. fastidiosa* CVC strain) it conferred no induction of *hxfA* in the *Xf*DSF-biosensor strain [8], thus it is still uncertain whether this fatty acid is a DSF molecule.

As part of our continued investigation into *C. sinensis*, *C. limonia* and their grafts to determine the chemical basis involved in the interaction between plant-*X. fastidiosa* and how the rootstock interferes in the metabolism of scion, we recently reported a screening by high performance liquid chromatography-ultraviolet spectroscopy (HPLC-UV) to check whether compounds from secondary metabolites were associated with the citrus infection. The data suggested that the flavonoids hesperidin and rutin, and the coumarins play a role in the plant-pathogen interaction [9]. We have now undertaken a further investigation of *X. fastidiosa* CVC strain (9a5c), in order to determine if diffusible signal factor was also present in the resulting bacterial supernatant and pellet volume after centrifugation. This bacterium is little known chemically.

## 2. Results

### 2.1. GC/MS Profiles of Hexane Fractions from X. fastidiosa 9a5c Culture Pellet and Supernatants Grown in PW Medium

Examination of the chromatograms of hexane extract from *X. fastidiosa* 9a5c culture pellet indicated the presence of over 18 major components, of which seven were identified. Qualitative identification consisted in the comparison of mass spectra recorded during the analysis and those contained in the instrument library (NIST107, NIST05, WILEY8) and with literature data [5,10]. Qualitative results were also shown in Figures S1 and S2. The identified constituents were 12-methyl- tetradecanoic acid C_15_H_30_O_2_ (Rt 11.50 min), 9-hexadecenoic acid C_16_H_30_O_2_ (Rt 12.27 min), hexadecanoate methyl ester C_17_H_34_O_2_ (Rt 12.73 min), 9-octadecenoic acid C_18_H_34_O_2_ (Rt 12.98 min), 13-methyl pentadecanoic acid C_16_H_32_O_2_ (Rt 13.41 min), 9-nonadecenoic acid C_19_H_36_O_2_ (Rt 19.39 min), 10-nonadecen-1-ol (Rt 29.37 min). Because the mass spectra of double-bond positional isomers and geometrical isomers are almost the same, some components which have isomers cannot be identified in this experiment, such as C16:9, C18:9, and C19:9. Methyl esters were identified by the *m*/*z* 74 fragment ion produced by the McLafferty rearrangement in which the Hγ migrates to the carboxyl group through a six-member transition state. However, methyl esters were obtained from pellet extracted with methanol stirring for 24 h, hence, they may not be a genuine natural product. 

The GC-MS analysis of hexane extract from *X. fastidiosa* 9a5c culture supernatant gave a direct and effective indication that the chemical molecules present were diketopiperazines, and the only exception referred to hexadecanoic acid (Rt 17.82 min) and 1-hexadecanol (Rt 32.17 min). The total ion chromatogram (TIC) is shown in Figure S3. Analysis was performed in full-scan mode and in selected reaction monitoring (SRM) mode. The amine fragments which arise from cleavage adjacent to both C=O are present in the mass spectra of all the diketopiperazines, using electron impact (70 eV) or electrospray ionization, and it is exemplified in Section 2.2. In symmetrical diketopiperazines, this fragmentation give identical amine fragments while in unsymmetrical diketopiperazines this cleavage yield two amine fragments. These two amine fragments constitute the most important diagnostic evidence for structure elucidation of diketopiperazines [11].

When the two peaks were of the same chemical substances but with different retention times in the chromatographic column, we supposed that one peak was for the d-amino acid and the other was for the l-isomer. The TIC and the mass spectra showed there were the following diketopiperazines present in the supernatant: cyclo(Val-Ala) (Rt 11.45 and 11.63 min), cyclo(Pro-Val) (Rt 13.74 and 14.48 min), cyclo(Pro-Leu/or Ile) (Rt 16.41 and 16.99 min), cyclo(Pro-Ile/or Leu) (Rt 17.30 and 17.42 min), cyclo(Val-Phe) (Rt 30.89 min), cyclo(Pro-Phe) (Rt 33.30 and 34.70 min). Unfortunately, in the overwhelming majority of instruments used for routine diketopiperazines analysis, such as GC-MS at 70 eV, leucine (Leu) and isoleucine (Ile) are indistinguishable. The major ions observed in the mass spectra are summarized in Table S1, and the numbers in total ion chromatogram in Figure S3 correspond to the diketopiperazines cited in this table.

Diketopiperazines can be generated by non-enzymatic cyclization of linear dipeptides at extremes of temperature or pH, and the complex medium used to culture *X. fastidiosa* 9a5c includes phytone peptone (enzymatic digest of soybean meal) and trypticase peptone (pancreatic digest of casein), which could produce cyclic peptides by heat sterilization [11]. Thus, we were concerned that they may have been generated by heat sterilization of this complex medium. 

The results from a negative control (PW medium ) show there are no obvious peaks at the retention times of diketopiperazines described previously (Figure S3). However, the HPLC-EI/MS in selected reaction monitoring (SRM) mode for the above diketopiperazines showed that a small amount of cyclo(Pro-Tyr), cyclo(Pro-Phe) and cyclo(Pro-Leu) or cyclo(Pro-Ile) had been generated in the culture medium, although the amount is too small to give a peak in the chromatogram (data not shown). This provides evidence that these three diketopiperazines are not only produced by *X. fastidiosa* 9a5c, but also in the medium by a chemical reaction. It is certain that the diketopiperazines formed by the medium is much less than those produced by the bacteria.

### 2.2. UFLC-MS/MS Profiles of Fraction from X. fastidiosa 9a5c Grown in XFM Medium

Diketopiperazines were long disregarded because many cyclodipeptides formed from protein hydrolysates were considered to be the byproducts of protein degradation [12]. Thus, to determine whether these diketopiperazines are either synthesized de novo from the products of primary metabolism or generated via the metabolism of bacterially generated peptides, *X. fastidiosa* 9a5c was grown in a simple defined medium (*X. fastidiosa* medium or XFM) containing a single carbon source and no preformed amino acids. Only three amino acids are used in this medium, l-glutamine, l-asparagine and l-cysteine, thus, diketopiperazines detected in *X. fastidiosa* 9a5c constituted by other amino acids would have been biosynthesized by the bacterium. The growth of *X. fastidiosa* in rich complex mediums as PD3 increase the number and rate of colony development, thus, this was also chosen to scale up bacterial growth and increase the metabolite production, to provide diketopiperazines as standards.

**Figure 1:**
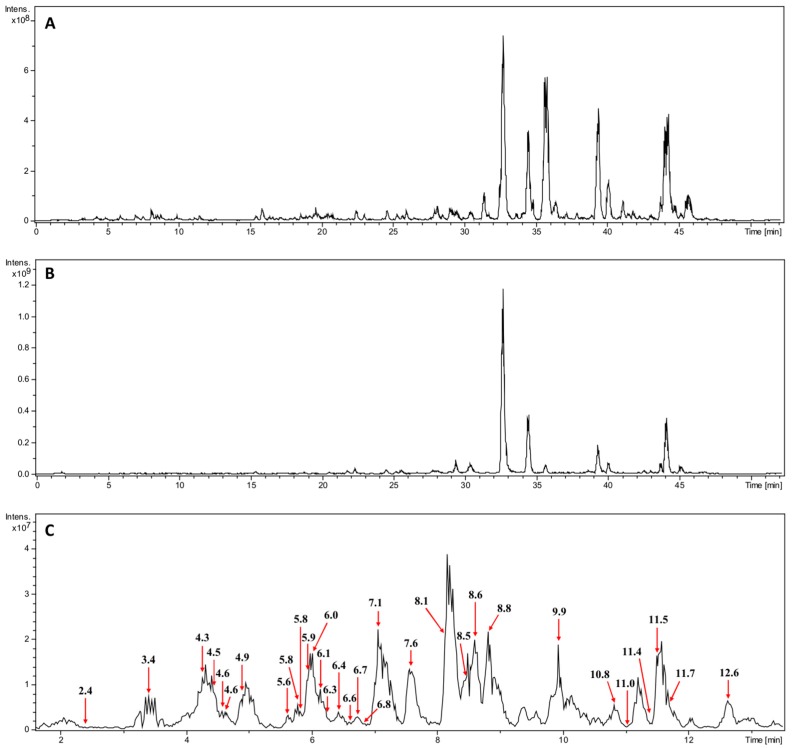
(**A**) The total ion chromatogram of acellular culture supernatant residues from *X. fastidiosa* 9a5c grown in XFM medium (UFLC-ESI-IT, in positive ion mode); (**B**) Negative control XFM medium; (**C**) The retention times of diketopiperazines were amplified.

Cells of *X. fastidiosa* were incubated in XFM liquid medium; however they had small dimensions compared with that observed in solid-medium culture in petri dishes. Thus, this part of the investigation focused on the extraction, separation and identification of the major compounds in *X. fastidiosa* 9a5c growth in solid medium XFM. Many analytical methods had been utilized for the analysis of diketopiperazines, and LC-ESI-MS had been shown to be a powerful technique for the analysis of these compounds due to its excellent ability in separation and identification. In this paper, a simple, rapid, and sensitive method, using Ultra-Fast Liquid Chromatography coupled with Electrospray Ionization ion trap mass spectrometry (UFLC-ESI-IT), was established for the identification of diketopiperazines in acellular culture supernatant residues. The result suggested that this technique could facilitate rapid and accurate identification of these compounds in *X. fastidiosa* 9a5c. We utilized knowledge of characteristic fragment ions of diketopiperazines to identify their structures [13]. The detailed total ion chromatogram (TIC) of acellular culture supernatant residues from *X. fastidiosa* 9a5c is shown in Figure 1.

**Scheme 1:**
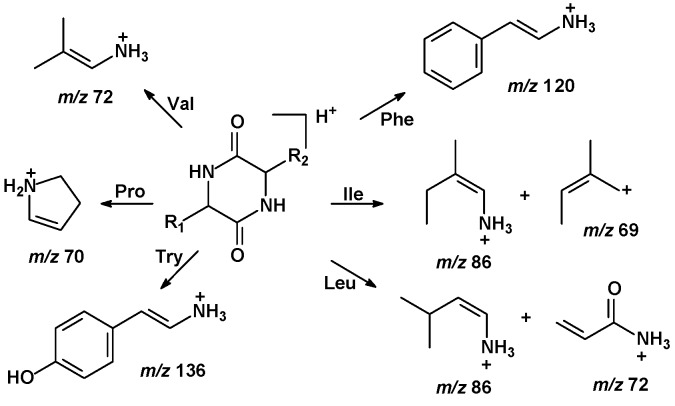
The amine fragments found as characteristic MS/MS product ions in the MS2 spectrum of the protonated diketopiperazines from *X. fastidiosa* 9a5c grown in XFM medium [13].

The amine fragments which arise from cleavage adjacent to both C=O are present in the mass spectra of all the diketopiperazines, and they constitute the most important diagnostic evidence for structure elucidation of these compounds in GC-MS or in ESI-MS/MS in positive mode. Scheme 1 shows the amine fragments found as characteristic MS/MS product ions in the MS2 spectrum of the protonated molecules of 21 structural isomers of diketopiperazines from *X. fastidiosa* 9a5c.

**Figure 2:**
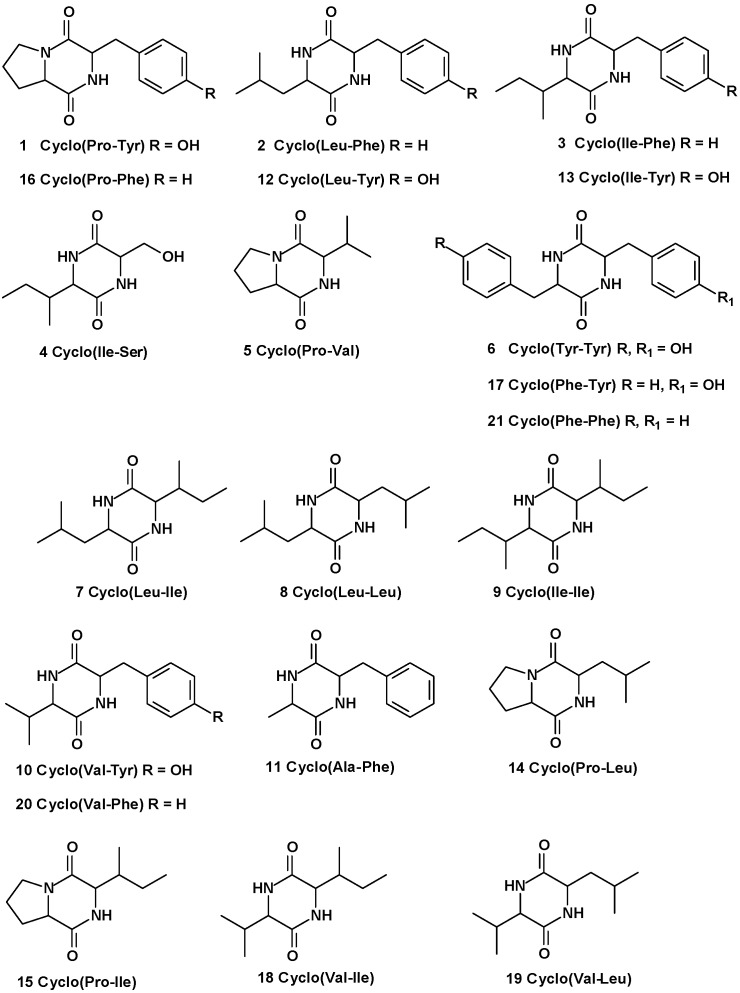
Structural isomers of diketopiperazines detected in acellular culture supernatant residues from *X. fastidiosa* 9a5c grown in XFM medium.

The main constituents are shown in Figure 2 and in Table 1 with the corresponding mass spectral characteristics. We became aware that different stereoisomers are present in the acellular culture supernatant. If we consider the configurations of amino acids, we can have four possible diketopiperazine stereoisomers (l/l-, d/d-, l/d-, d/l-). The presence of various stereoisomers in bacterial cultures can be attributed to the natural use of l- and d-amino acids by microorganisms, or to epimerization. We observed the four possible stereoisomers of cyclo(Leu-Phe)/or cyclo(Ile-Phe) in the bacterial extracts (Table 1, *m/z* 261).

The ion at *m/z* 86 (C_5_H_12_N) was characteristic of the amino acid residue Ile/Leu. Ile and Leu had similar product profiles, in which the only different product ion had an *m/z* value of 69 (C_5_H_9_) for Ile instead of 72 (C_3_H_6_NO) in the case of Leu. Unfortunately, in the UFLC-ESI-IT instrument used for diketopiperazines analysis, leucine and isoleucine were also indistinguishable. Thus, it would be possible to observe at least nine stereoisomers [cyclo(d-Leu-d-Leu), cyclo(l-Leu-l-Leu), cyclo(d-Leu-l-Leu), cyclo(d-Ile-d-Ile), cyclo(l-Ile-l-Ile), cyclo(d-Ile-l-Ile), cyclo(d-Leu-d-Ile), cyclo(l-Leu-l-Ile), cyclo(d-Leu-l-Ile)]. We observed six of the possible stereoisomers (Table 1, *m/z* 227).

All diketopiperazines isolated were absent from extracts of uninoculated solid medium XFM. In addition, diketopiperazines biosynthesised from amino acids used in XFM medium were not found in *X. fastidiosa* 9a5c supernatants, thus we can argue that the above compounds must be naturally occurring in the bacterium. Diketopiperazines are reported for the first time from *X. fastidiosa*, however, all those detected in this study have been recorded previously from the bacteria, fungi, algae, plants and animals (Table 1 and [20]).

**Figure 3:**
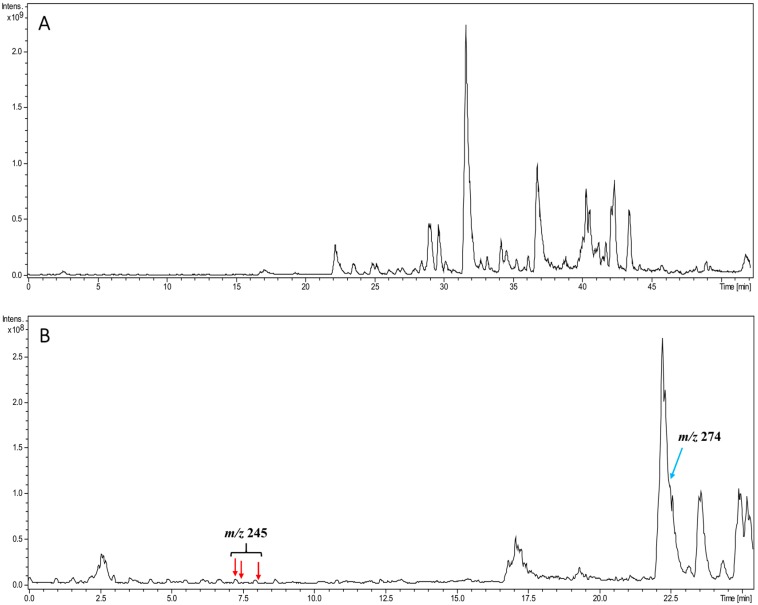
(**A**) The total ion chromatogram of culture pellet residues from *X. fastidiosa* 9a5c grown in XFM medium (UFLC-ESI-IT, in positive ion mode); (**B**) The retention times of dipeptides were amplified.

As already mentioned, the analysis was also performed on *X. fastidiosa* 9a5c culture pellet, and these compounds were lacking in this fraction. Examination of the total ion chromatogram (TIC) of methanol extract from pellet (Figure 3) indicated the presence of dipeptides (Rt 7.2, 7.4, 8.1, *m/z* 245; Rt 22.5 min *m/z* 274; Figures S4 and S5) and fatty acids.

The dipeptide stereoisomers (Rt 7.2, 7.4, 8.1, *m/z* 245) isoleucyl-leucine, or leucyl-isoleucine, or isoleucyl-isoleucine, or leucyl-leucine were supported by the mass spectrum which showed fragments at *m/z* 86 (C_5_H_12_N, 100%) characteristic of the amino acid residue Ile/Leu (Figure 2 and Figure S4, Scheme S1). However, while all the other fragments confirm the structural proposal, unfortunately they do not allow determining which of the two amino acids would be present, if they are distinct and which one contains the amide group. Therefore the three stereoisomers could correspond to the following structural isomers: isoleucyl-leucine, or leucyl-isoleucine, isoleucyl-isoleucine, or leucyl-leucine. The mass spectrum for the other dipeptide gave significant fragments for *m/z* 88 [C_3_H_10_N_3_; CH_3_CH_2_NHC=N^+^H_2_(NH_2_)] and 102 [C_5_H_12_NO; CH_3_CH(CH_3_)CH(NH_2_)CH=O^+^H] requiring the presence of arginine and valine (Figure S5; Scheme S2). The fragment *m/z* 102 suggested that the structure of dipeptide is valyl-arginine. The biosynthesis of dipeptides may occur by direct interaction of the respective amino acids, thus both isolated compounds are not artifacts formed from XFM medium, since the amino acids isoleucine, leucine, valine and arginine, and protein were not used in this medium. Cyclization could have occurred in isoleucyl-leucine dipeptide intermediate, leading to the release of the cyclic dipeptide cyclo(Ile-Leu) in solution, since the diketopiperazine was detected only in acellular culture supernatant residues. As noted supernatant residues appear to be more prolific in the production of cyclic dipeptides, and relatively poor in dipeptides, suggesting that the latter are cyclized to diketopiperazines and released into the medium to be used as quorum-sensing, or as antifungal and antibacterial agents.

Mass spectral and retention time index libraries for GC-MS, as the currently leading and widely accepted NIST mass spectral search program, perform qualitative analysis of any biological sample and allows unequivocal metabolite identification. NIST11.0 provide libraries comprising many fatty acids, thus in order to identify this class of compounds in *X. fastidiosa* 9a5c culture pellet, this was also analyzed by GC-MS. Examination of the chromatogram (Figure S6) and the mass spectra (Figure S7) indicated the presence of fatty acids, of which 5 were identified as pentadecanoic acid (*m/z* = 242; C_15_H_30_O_2_; Rt = 13.92 min), hexadecanoic acid methyl ester (*m/z* = 270; C_17_H_34_O_2_; Rt = 15.13 min), oleic acid [(*Z*)-octadec-9-enoic acid] (*m/z* = 282; C_18_H_34_O_2_; Rt = 15.39 min), hexadecanoic acid (*m/z* = 256; C_16_H_32_O_2_; Rt = 15.83 min), and octadecanoic acid (*m/z* = 284; C_18_H_36_O_2_; Rt = 17.63 min). Methyl esters were obtained from pellet extracted with methanol, thus, they may not be a genuine natural product.

## 3. Discussion

Beaulieu and collaborators [6] previously isolated 2-*cis*-tetradecenoic acid from a grape strain of *X. fastidiosa*, which was biologically active in the *Xf*DSF-biosensor strain [7]. Diffusible signal factor (DSF) synthase RpfF of *Xylella fastidiosa* is a unique crotonase that has both 3-hydroxyacyl-acyl carrier protein (ACP) dehydratase and thioesterase activity. RpfF first catalyzes the formation of a double bond between carbons 2 and 3 of a 3-hydroxyacyl moiety and then hydrolyzes the thioester bond with ACP to release a free acid. The DSF sensing mechanism in *X. fastidiosa*, unlike in *X. campestris*, is RpfF dependent; an *rpfF* deletion mutant could not sense externally applied DSF, while a strain harboring an *rpfF* variant (designated rpfF*) in which DSF synthesis was blocked via substitution of two glutamic acid residues with alanine residues (E141A and E161A), was able to sense and respond to DSF. This strain was the basis for an *X. fastidiosa* based DSF sensor, which the authors designate the *Xf*DSF-biosensor strain.

Recently, Ionescu and collaborators [8] showed that *X. fastidiosa* produces a particularly large variety of similar, relatively long-chain-length 2-enoic acids that are active in modulating gene expression. *X. fastidiosa* RpfF (*Xf*RpfF) is also capable of producing a variety of both saturated and unsaturated free fatty acids. However, only 2-*cis* unsaturated acids were found to be biologically active in *X. fastidiosa*. This bacterium is particularly responsive to a novel DSF species, 2-*cis*-hexadecenoic that the authors term *Xf*DSF2. It is also responsive to other, even longer 2-enoic acids to which other taxa such as *Xanthomonas campestris* are unresponsive. The 2-enoic acids that are produced by *X. fastidiosa* are strongly affected by the cellular growth environment, with *Xf*DSF2 not detected in culture media in which 2-tetradecenoic acid (*Xf*DSF1) had previously been found. *X. fastidiosa* is responsive to much lower concentrations of *Xf*DSF2 than *Xf*DSF1 [8]. Ionescu and collaborators [8] reported that 12-methyltetradecanoic acid (CVC-DSF) and other saturated fatty acids that do not activate *hxfA* or other DSF-responsive genes in *X. fastidiosa* antagonize DSF-mediated signaling in *X. fastidiousa*. This antagonism is apparently not associated with any toxicity to the cells and thus growth inhibition. Instead, such saturated molecules appear to compete directly with 2-enoic acids for DSF receptors such as RpfC, since the responses to various enoic acids were reduced in a dose-dependent manner by an equal concentration of such molecules. This signaling antagonism did not appear to be very specific, as the response to all enoic acids investigated could be blocked by a given saturated fatty acid, and a given saturated fatty acid, such as palmitic acid, could block the response to more than one enoic acid, such as *Xf*DSF, *Xf*DSF2, and palmitoleic acid.

All the above work in *X. fastidiosa* Rpf system was developed with a grape strain; however there has not been any extensive study on Rpf in a CVC strain. CVC-DSF 12-methyltetradecanoic acid was obtained *X. fastidiosa* strain 9a5c acellular culture supernatant grown in PW liquid medium [5]. We report here other six fatty acids from *X. fastidiosa* CVC strain (9a5c) culture pellet, while culture supernatant produced many diketopiperazines, and only one fatty acid (hexadecanoic acid). *X. fastidiosa* CVC strain (9a5c) in our study was also grown in PW liquid medium, and it should have revealed 12-methyltetradecanoic acid, if it had been present. However, Simionato et al. [5] comment that CVC-DSF was tentatively identified as 12-methyltetradecanoic acid. The component more abundant in hexane extract from culture pellet was 13-methylpentadecanoic acid C_16_H_32_O_2_ (Rt 13.41 min), and this appears to be the first record of this fatty acid from *X. fastidiosa* CVC strain (9a5c), and it was not found in Pierce’s disease *X. fastidiosa*. Hexadecanoic acid was the only one found in both cultures pellet (medium XFM) and supernatant (PW liquid medium), and it was the only one obtained in Pierce’s disease (PD) *X. fastidiosa* [8]. The composition of the mixed DSF signals produced by *X. fastidiosa* appears to be influenced by the composition of the culture media in which it grew. *X. fastidiosa* 9a5c culture pellet growth in solid medium XFM produced 3 fatty acids different of those obtained from the bacterium grown in PW liquid medium, only hexadecanoic acid was found in both. In addition, hexadecanoic and octadecanoic acids occur in CVC-*X. fastidiosa* in solid medium XFM and PD-*X. fastidiosa*. Isolation procedures used in this study should have revealed long-chain-length 2-enoic acids if they had been present. However, it is premature to use absence of this class of fatty acid as an argument that CVC-*X. fastidiosa* has different DSF-mediate quorum sensing. Clearly much more detailed investigations of CVC-*X. fastidiosa* will be essential for a better understanding of its fatty acid-based quorum sensing.

Many scientists are perhaps interested in fatty acids from *X. fastidiosa*, and will rarely identify all of the potentially active important classes of compounds present in the bacterium, e.g., diketopiperazines. Thus, clearly *X. fastidiosa* diketopiperazines deserve more attention than they have received so far. Studies of ergot alkaloid biosynthesis in the fungus *Claviceps purpurea* have shown that the d-lysergyl peptide synthetase (LPS), which is responsible for the formation of the d-lysergyl peptide-diketopiperazine, an intermediate in the biosynthesis of ergopeptines, is devoid of a C-terminal thioesterase domain. LPS binds d-lysergic acid (alkaloid skeleton) and three amino acids (e.g., alanine, phenylalanine and proline for ergotamine alkaloid) as thioesters in an ATP-dependent reaction. This resembles the non-ribosomal protein thiol-template systems. Diketopiperazine cyclization is thus thought to occur spontaneously, leading to the release of the intermediate in solution [22]. Such spontaneous cyclization could also been occurring in *X. fastidiosa* 9a5c for the isoleucyl-leucine dipeptide intermediate to cyclo(Ile-Leu) biosynthesis.

However, Lautru and collaborators [23] used the cyclic dipeptide oxidase (CDO) peptide sequence information to isolate a 3.8 kb *Streptomyces noursei* DNA fragment that directs albonoursin [cyclo(ΔPhe-ΔLeu)] biosynthesis in *S. lividans*. This fragment encompasses four complete genes: *albA* and *albB*, necessary for CDO activity; *albC*, sufficient for cyclic dipeptide precursor formation, although displaying no similarity to nonribosomal peptide synthetase (NRPS) genes; and *albD*, encoding a putative membrane protein. Therefore, as AlbD is not strictly necessary for albonoursin biosynthesis, the authors proposed that it is involved in the transport of albonoursin across membranes or serves as a target for albonoursin acting in the same way as other small diffusible molecules in intercellular communication.

In order to determine the role of diketopiperazines in Gram-negative bacteria, much work has been done. Degrassi et al. [24] found four different diketopiperazines [cyclo(l-Tyr-l-Pro), cyclo(l-Leu-l-Pro), cyclo(l-Phe-l-Pro), and cyclo(l-VaL-l-Leu)] in *Pseudomonas putida* WCS358, and found that two of the four synthetic DKPs cyclo(l-Tyr-l-Pro) and cyclo(l-Leu-l-Pro) activated *N*-acylhomoserine lactones (AHL)-biosensor *Agrobacterium tumefaciens* NT1(pDCI41E33) while the others activated AHL-biosensor *Escherichia coli* JM109 (pSB401). *N*-acylhomoserine lactones (the signal molecules characteristic of Gram-negative bacteria) differing in their length and degree of saturation of the acyl chain. These results are in accordance with those previously reported by Holden et al. [25] for cyclo(l-Phe-l-Pro) and cyclo(l-Leu-l-Pro), except that Holden found cyclo(l-Tyr-l-Pro) can activate *E. coli* JM109(pSB401). Thus, it was observed that some of the diketopiperazines activate certain AHL-biosensors. Thereby, Holden postulated that diketopiperazines may be a novel signal molecule of Gram-negative bacteria or interact with AHL-based quorum sensing. In addition, diketopiperazines were classified as signal molecules by Barnard and Salmon in 2004 [26]. However, the role of these molecules in signal transduction still remains largely unknown.

All diketopiperazines isolated from *X. fastidiosa* were previously proved to activate the quorum sensing biosensors of other microorganisms. However, the absolute configuration of the diketopiperazines must be important for the intercrossing communication. Thus, we consider that biosynthetic studies using optically pure amino acids will provide better information on the role of diketopiperazines. Unfortunately, these molecules are able to epimerize; therefore, this will be a difficult challenge, which will require synthesis of optically pure diketopiperazines and control of their epimerization. Only a few diketopiperazine biosynthetic pathways that belong to either nonribosomal peptide synthetases NRPS-dependent pathways or cyclodipeptide synthases CDPS-dependent pathways have been fully characterized [12,23]. For each of these pathways, the nature of the diketopiperazines produced has been identified and its mechanism of formation has been elucidated. It is conceivable that, in some pathogenic fungi and bacteria, diketopiperazines play an essential role in virulence. Therefore, every step of the biosynthesis might be an appropriate drug target for antifungal or antibacterial therapy. Once the genome of the producing microbe is sequenced and the pathways are mined, as in *X. fastidiosa*, there will be more opportunities to clone, overexpress, and purify these enzymes to test on substrates with the help of synthetic chemists. Thereby, biosynthetic experiments for diketopiperazines in *X. fastidiosa* are in progress.

## 4. Materials and Methods

### 4.1. Bacterial Strain 

The *Xylella fastidiosa* CVC strain used in this study was the 9a5c [2] obtained from the Centro APTA Citros “Sylvio Moreira”, Cordeirópolis, SP, Brazil.

### 4.2. Preparation of X. fastidiosa Samples Grown in PW Medium for GC-MS Analysis

*Citrus sinensis* plants were inoculated with strain 9a5c of *X. fastidiosa* and when CVC symptoms were visible, petioles and stems were collected and aseptically ground in PBS (phosphate *buffered* saline). The suspension was distributed on plates containing periwinkle wilt (PW) medium [27]. The first colonies were observed between 10 to 15 days after plating. To obtain cells in biofilm, several individual colonies were transferred to a polypropylene tube containing 3 mL of PW broth. When the OD600 nm reached 0.3, the tubes were vortexed and the cells transferred to a 1 L flask containing 300 mL of PW broth, previously described to promote *X. fastidiosa* biofilm formation in vitro [28]. A total of three flasks were used in each analysis. After three days of growth at 28 °C in a rotary shaker at 120 rpm a thin biofilm formation was observed attached to the glass at the medium-air interface. The sample was collected after 20 days when the most abundant layer of biofilm formation was observed. The biofilm layer was scraped from flask to liquid phase and it was centrifuged at 8000× *g* for 5 min at 28 °C. In each precipitate (pellet) was added methanol, and allowed under moderate magnetic stirring for 3 × 24 h, and then it was filtered by gravity into a flask. The methanol was evaporated under reduced pressure to afford a residue. The concentrated MeOH was partitioned into hexane, and MeOH. After the biofilm layer was scraped and centrifugation, the liquid phase was partitioned with ethyl acetate, and the organic solvent was further concentrated under vacuum to give the crude extract. The concentrated EtOAc was partitioned into hexane, and EtOAc. Both hexane extracts (*X.f.*9a5c PWpellet; *X.f.*9a5c PWsupernatant) were used in GC-MS experiments. The same method was used to extract chemicals from the culture without bacteria *X. fastidiosa* 9a5c as a negative control (PWnc).

The analysis were carried out on a GC-17A gas chromatograph (Shimadzu, Kyoto, Japan) fitted with a fused silica AT-5ms (30 m × 0.25 mm ID, 0.25 μm film thickness) capillary column with helium as the carrier gas at a flow rate of 1.2 mL min^−1^. The temperature was programmed initially at 150 °C and then increased with a rate of 5 °C min^−1^ to 280 °C, isotherm for 10 min. The injection was split and its temperature was 250 °C. The interface temperature was 280 °C. Injection volume was 1.0 μL solution in ethyl acetate. The chromatograph was coupled to a Shimadzu QP5000 mass selective detector at 70 eV. Scanning speed was 0.5 scan s^−1^ from *m/z* 50 to 500. Identification of the components was made by determination of their retention indices relative to those of a homologous series of *n*-alkanes (C_10_−C_24_) [29], by comparison with fragmentation patterns in mass spectra with those stored on the spectrometer database and bibliography [30].

### 4.3. Preparation of X. fastidiosa Samples 9a5c Grown in XFM Medium for UFLC-MS/MS and GC-MS Analysis

Strain 9a5c preserved in periwinkle wilt GelRite or PWG medium were grown on biochemical oxygen demand (B.O.D) at 28 °C for 7 days on PWG solid medium [27,31]. Cultured cells were obtained from PWG agar (63 plates) by removing colonies with a loop and then transferred into a Falcon tube containing 12 mL of PBS, which were then homogenized by vortex agitation. Subsequent subcultures were made by transferring 40 µL of the broth culture to 200 plates with a defined medium *X. fastidiosa* medium or XFM (1.5 g L^−1^ of trisodium citrate, 1.5 g L^−1^ of disodium succinate, 1.0 g L^−1^ of K_2_HPO_4_, 1.0 g L^−1^ of KH_2_PO_4_, 0.5 g L^−1^ of MgSO_4_, 3.0 g L^−1^ of l-glutamine, 1.0 g L^−1^ of l-asparagine, 0.5 g L^−1^ of l-cysteine, 10 mL of hemin chloride and 8 g L^−1^ of Gelzan™ (gellan gum) [32]. The plates were incubated on B.O.D at 28 °C for 16 days in continuous darkness. After 16 days cultured cells were obtained from XFM medium by removing colonies with a loop and then transferred into two Eppendorf tubes containing 1 mL of sterile distilled water, which were then three times centrifuged at 8000× *g* for 15 min at 4 °C. The precipitate (pellet) was transferred to beaker flask containing 20 mL of MeOH and extractions were performed three times in an ultrasound bath for 60 s. Subsequently, the flask was allowed to stand for 3 h and then it was centrifuged at 8000× *g* for 10 min at 4 °C. The MeOH phase was evaporated under reduced pressure to provide pellet residue (*X.f.*9a5c XFMpellet) for Ultra-Fast Liquid Chromatograph-mass spectrometry (UFLC-MS) experiments. After the cultured cells were obtained from XFM medium and centrifugation, the liquid phase was evaporated under reduced pressure to provide acellular culture supernatant residue (*X.f.*9a5c XFMsupernatant) for UFLC-MS experiments. 

A similar method was used to extract chemicals from XFM medium without bacteria *X. fastidiosa* 9a5c as a negative control. XFM medium was transferred to beaker containing 1.4 L of EtOAc and extractions were performed three times in an ultrasound bath for 5 min. Subsequently, the flask was allowed to stand for 24 h and then filtered by gravity. The EtOAc was then evaporated under reduced pressure to provide XFM medium (negative control) residue (XFMnc) for UFLC-MS experiments.

*Xylella fastidiosa* 9a5c was also grown in modified Pierce Disease medium (PD3) to increase the number and rate of colony development and consequently, to obtain diketopiperazines in higher concentrations. As above subcultures were made by transferring 40 µL of the broth culture to 100 plates with a PD3 medium (4.0 g L^−1^ of tryptone, 2.0 g L^−1^ of soytone, 1.0 g L^−1^ of trisodium citrate, 1.0 g L^−1^ of disodium succinate, 2.0 g L^−1^ of potato starch, 1.5 g L^−1^ of K_2_HPO_4_, 1.0 g L^−1^ of KH_2_PO_4_, 1.0 g L^−1^ of MgSO_4_. 7H_2_O, 10 mL of hemin chloride, and adjust to pH 6.8, and 15 g L^−1^ agar) [33].

The above method was used to extract chemicals from the PD3 culture with and without bacteria *X. fastidiosa* 9a5c, yielding pellet residue (*X.f.*9a5c PD3pellet), acellular culture supernatant residue (*X.f.*9a5c PD3supernatant) and PD3 medium (negative control) residue (PD3nc) for UFLC-MS experiments.

#### 4.3.1. UFLC-MS Parameters

*X.f.*9a5c XFM pellet (3.3 mg mL^−1^), *X.f.*9a5c XFM supernatant (2.1 mg mL^−1^), XFMnc (1.9 mg mL^−1^), *X.f.*9a5c PD3 pellet (3.9 mg mL^−1^), *X.f.*9a5c PD3 supernatant (3.6 mg mL^−1^), and PD3nc (2.6 mg mL^−1^) was dissolved in a 1:1 mixture of Milli-Q H_2_O and MeOH. The solution was filtered through a millipore filter of 0.22 µm before UFLC analysis. 

A Shimadzu LC-20AD series Ultra-Fast Liquid Chromatograph (UFLC) system, with photodiode array detector (DAD, Shimadzu SPD-M20A) was employed for chromatographic separation using a Kinetex XB-C18 column (100 mm × 2.1 mm, 2.6 μm) (Phenomenex, Torrance, CA, USA) at a flow rate of 250 µL min^−1^, 20 µL of extracts were injected in each analysis. The mobile phase consisted of a water/CH_3_CN gradient both with 0.1% of formic acid. The percentage of organic modifier (B) was changed linearly as follows: 0 min, 8%; 25 min, 60%; 45 min, 98%; 50 min, 98%. The column temperature was set to 35 °C. Shimadzu UFLC system was connected to an AmaZon SL Bruker^®^ (Billerica, MA, USA) ion trap mass spectrometer operating at positive and negative electrospray ionization mode. The analyses were carried out using nitrogen as the nebulizing (40 psi) and drying gas (9 L/min, 250 °C), and argon as collision gas. The capillary voltage was set at 2.8 kV. The data was analyzed by Data Analysis (Analysis 4.3, Bruker Daltonics, Boston, MA, USA) software.

#### 4.3.2. GC-MS of Culture Pellet Residue from *X. fastidiosa* 9a5c Grown in XFM Medium

Culture pellet residue from *X. fastidiosa* 9a5c grown in XFM medium was obtained as above for GC-MS analysis, which was performed using capillary gas chromatography (GC) connected to an electron ionization mass spectrometric detector (EI-MS; GC-2010 equipped with a GCMS-TQ8030; at 70 eV, Shimadzu) with a RTx-5 MS column (5% diphenyl and 95% dimethylpolysiloxane; 30 m × 0.25 mm i.d × 0.25 µm film thickness). Helium was used as carrier gas. The carrier gas inlet pressure was regulated for a constant flow of 1.4 mL min^−1^. GC was carried out with a temperature-programmed injection as follows: 150 °C raised by 5 °C min^−1^ to 280 °C, and isotherm for 10 min. The injection was split and its temperature was 250 °C. The interface temperature was 280 °C. Injection volume was 1.0 μL solution in methanol. Scanning speed was 0.3 scan s^−1^ from *m*/*z* 50 to 700. Identification of the components was made by determination of their retention indices relative to those of a homologous series of *n*-alkanes (C_10_−C_24_) [29], by comparison with fragmentation patterns in mass spectra with those stored on the spectrometer database (the NIST Mass Spectral Library 11.0) and bibliography [30].

## 5. Conclusions

Use of the simple defined *X. fastidiosa* medium (XFM) allowed us to confirm that diketopiperazines are synthesized de novo from the products of primary metabolism in *X. fastidiosa*. The present UFLC-MS/MS method was simple, rapid, and sensitive to detect diketopiperazines for the first time from *X. fastidiosa.* GC/MS method also showed to be accurate, viable and satisfactory for the determination of fatty acids in this bacterium, which were not biologically active as diffusible signal. In addition, the role of diketopiperazines in signal transduction still remains unknown. In order to determine the role of these compounds in *X. fastidiosa* further plant-pathogen interaction and biosynthesis experiments are in progress.

## Figures and Tables

**Table 1:** Compounds identified by UFLC-ESI-IT in acellular culture supernatant residues from *X. fastidiosa* 9a5c grown in XFM medium.

Structural Isomers/Stereoisomers	Rt ** (min)	M.W. *	*m*/*z* (%) Ions	Reference
**1** Pro-Tyr				
cyclo(Pro-Tyr)	2.4	261	233(100); 181(69); 63(8); 136(7)	[14,15,16,17]
cyclo(Pro-Tyr)	4.3	261	233(96); 216(11); 197(17); 155(12); 147(20); 136(100); 119(4)	[14,15,16,17,18]
**2** Leu-Phe				
**3** Ile-Phe				
cyclo(Leu-Phe)	5.6	261	243(35); 233(100); 215(99); 187(23); 170(8); 120(84); 103(4); 86(21)	[13,14,16,18,19]
cyclo(Ile-Phe)				
cyclo(Leu-Phe)	6.1	261	243(100); 233(57); 216(11); 188 (8); 131(7); 120(89); 86 (39)	[13,14,16,18,19]
cyclo(Ile-Phe)				
cyclo(Leu-Phe)	11.0	261	243(11); 233(100); 216(47); 188(4); 162(3); 120(77); 86(12)	[13,14,16,18,19]
cyclo(Ile-Phe)				
cyclo(Leu-Phe)	11.5	261	233(100); 216(29); 188(3); 120(74); 86(13)	[13,14,16,18,19]
cyclo(Ile-Phe)				
**4** Ile-Ser				
cyclo(Ile-Ser)	3.4	201	183(27); 173(100); 115(4); 86(4)	[19]
**5** Pro-Val				
cyclo(Pro-Val)	4.5	197	180(17); 169(100); 152(8); 141(17); 124 (15); 72(18); 70(13)	[13,14,16,18,20]
**6** Tyr-Tyr				
cyclo(Tyr-Tyr)	4.6	327	299(100); 282(7); 254(3); 221(19); 136(39); 107(4)	[21]
**7** Leu-Ile/**8** Leu-Leu				
**9** Ile-Ile				
cyclo(Leu-Ile)	4.6	227	209(13); 199(41); 181 (44); 166 (3); 153 (6); 86(100)	[18,19,21]
cyclo(Leu-Leu)				
cyclo(Ile-Ile)				
cyclo(Leu-Ile)	4.7	227	209(14); 199(46); 181 (30); 166 (3); 153 (5); 136(10); 129(4); 114(5); 86(100); 70(3); 69(1)	[18,19,21]
cyclo(Leu-Leu)				
cyclo(Ile-Ile)				
cyclo(Leu-Ile)	4.9	227	209(12); 199(100); 181(36); 171(5); 153(28); 136(21); 86(92); 68(4)	[18,19,21]
cyclo(Leu-Leu)				
cyclo(Ile-Ile)				
cyclo(Leu-Ile)	6.4	227	209(15); 199(31); 181 (15); 153 (7); 142(4); 114(12) 86(100)	[18,19,21]
cyclo(Leu-Leu)				
cyclo(Ile-Ile)				
cyclo(Leu-Ile)	10.8	227	199(75); 182(100); 154(6); 86(37)	[18,19,21]
cyclo(Leu-Leu)				
cyclo(Ile-Ile)				
cyclo(Leu-Ile)	11.4	227	199 (28); 182(100); 154(5); 86(17)	[18,19,21]
cyclo(Leu-Leu)				
cyclo(Ile-Ile)				
**10** Val-Tyr				
cyclo(Val-Tyr)	5.8	263	235(100); 218(7); 190(8); 157(4); 136(71)	[13,14,16]
**11** Ala-Phe				
cyclo(Ala-Phe)	5.8	219	191(100); 174(8); 120(68)	[14,16,21]
cyclo(Ala-Phe)	6.0	219	191(100); 174(10); 120 (71)	[14,16,21]
**12** Leu-Tyr				
**13** Ile-Tyr				
cyclo(Leu-Tyr)	5.9	277	249(100); 232(8); 171 (11); 147(4); 136 (60); 86(5)	[13,19]
cyclo(Ile-Tyr)				
cyclo(Leu-Tyr)	6.6	277	260 (24); 258(25); 249(100); 241(4); 232(21); 204(12); 171(25); 164(5); 147(3); 136(98); 119(3); 107(37); 86(9)	[13,19]
cyclo(Ile-Tyr)				
cyclo(Leu-Tyr)	6.7	277	249(100); 232(24); 204(3); 171(12); 136(52)	[13,19]
cyclo(Ile-Tyr)				
**14** Pro-Leu				
**15** Pro-Ile				
cyclo(Pro-Leu)	6.3	211	194(33); 183(100); 166(20); 155 (41); 138(16); 86(16); 70 (22)	[13,14,16,18,20]
cyclo(Pro-Ile)				
cyclo(Pro-Leu)	6.8	211	183(100); 166(12); 155(34); 138(12); 127(8); 114(17); 86(16)	[13,14,16,18,20]
cyclo(Pro-Ile)				
cyclo(Pro-Leu)	7.1	211	194(35); 183(100); 166(23); 155(22); 138(12); 127(7); 114(3); 86(62); 70(68)	[13,14,16,18,20]
cyclo(Pro-Ile)				
**16** Pro-Phe				
cyclo(Pro-Phe)	7.6	245	217(100); 200 (5); 172 (3); 131(3); 120 (43); 70 (25)	[13,14,16,18,20]
cyclo(Pro-Phe)	8.1	245	217(100); 200(3); 189(3); 120(90); 70 (40)	[13,14,16,18,20]
**17** Phe-Tyr				
cyclo(Phe-Tyr)	8.5	311	283(100); 266(4); 238(9); 205 (12); 149(3); 136(41); 120 (16)	[13]
**18** Val-Ile				
**19** Val-Leu				
cyclo(Val-Ile)	8.6	213	185(100); 168(40); 140(9); 86(8); 72(9)	[16,18]
cyclo(Val-Leu)				
cyclo(Val-Ile)	8.8	213	195(3); 185(97); 168(100); 157(4); 140(11); 86(14); 72(20)	[14,16,18]
cyclo(Val-Leu)				
**20** Val-Phe				
cyclo(Val-Phe)	9.9	247	219(100); 202(19); 174(8); 120 (68)	[13,14,16,18]
**21** Phe-Phe				
cyclo(Phe-Phe)	11.7	295	267(100); 250(18); 239(4); 207(5); 154(5); 120 (70)	[18,21]
cyclo(Phe-Phe)	12.6	295	267(99); 250(7); 120 (100)	[18,21]

* M.W. Molecular Weight; ** Rt. Retention time; unfortunately by UFLC-ESI-IT, leucine and isoleucine were indistinguishable; the absolute configurations of diketopiperazines have not been determined.

## Supplementary Materials

The following are available online: Figures S1–S7, Schemes S1–S2, and Table S1.
