# Supplementary material for: A Simple Defined Medium for the Production of True Diketopiperazines in Xylella fastidiosa and Their Identification by Ultra-Fast Liquid Chromatography-Electrospray Ionization Ion Trap Mass Spectrometry
Source: Molecules. 2017 Jun 13;22(6):985. doi: 10.3390/molecules22060985 (PMC6152636; doi:10.3390/molecules22060985)
Supplement: Supplementary file 1 [file molecules-22-00985-s001.pdf]

Supplementary Materials

A Simple Defined Medium for the Production of True Diketopiperazines in *Xylella fastidiosa* and their Identification by Ultra-Fast Liquid Chromatography-Electrospray Ionization Ion Trap Mass Spectrometry

Michelli Massaroli da Silva [1], Moacir dos Santos Andrade [1], Anelize Bauermeister [2], Marcus Vinícius Merfa [3], Moacir Rossi Forim [1], João Batista Fernandes [1], Paulo Cezar Vieira [1], Maria Fátima das Graças Fernandes da Silva [1,*], Norberto Peporine Lopes [2], Marcos Antônio Machado [3], Alessandra Alves de Souza [3]

[1] Departamento de Química, Universidade Federal de São Carlos, CP 676, 13565-905 São Carlos - SP, Brazil; mimassaroli@gmail.com (MMS); msandrade2003@gmail.com (MSA); mrforim@yahoo.com.br (MRF); djbf@ufscar.br (JBF); paulo@dq.ufscar.br (PCV); dmfs@ufscar.br (MFGFS)

[2] Núcleo Pesquisas em Produtos Naturais e Sintéticos, Faculdade de Ciências Farmacêuticas de Ribeirão Preto, Universidade de São Paulo, 14040-903, Ribeirão Preto, SP, Brazil; ane_qui@hotmail.com (AB); npelopes@fcfrp.usp.br (NPL)

[3] Centro APTA Citros Sylvio Moreira, Instituto Agronômico, CP 04,13490-970 Cordeirópolis, - SP, Brazil; marcussilva727@uol.com.br (MVM); marcos@centrodecitricultura.br (MAM); alessandra@centrodecitricultura.br (AAS)

* Correspondence: dmfs@ufscar.br; Tel.: + 55 (16) 3351.8093

Table S1. Compounds identified by GC-MS in the hexane extract from *X. fastidiosa* 9a5c culture supernatant grown in PW medium.

Compounds	Peak	M.W.*	Rt** (min)	*m/z* (%) ions
cyclo(Val-Ala)	5	168	11.45	128 (100), 70 (60), 55 (38), 86 (32), 113 (32)
cyclo(Val-Ala)	6	168	11.63	128 (100), 113 (38), 99 (25), 57 (22), 149 (20)
cyclo(Pro-Val)	8	196	13.74	70 (100), 154 (58), 125 (28), 72 (30), 55 (25)
cyclo(Pro-Val)	9	196	14.48	70 (100), 154 (50), 125 (42), 68 (18), 55 (15)
cyclo(Pro-Leu)	10	211	16.41	70 (100), 154 (75), 86 (26), 125 (22), 68 (20)
cyclo(Pro-Leu)	11	211	16.99	70 (100), 154 (70), 86 (28), 55 (12), 125 (20)
cyclo(Pro-Ile)	12	211	17.30	70 (100), 154 (55), 125 (23), 55 (23), 86 (18)
cyclo(Pro-Ile)	13	211	17.42	70 (100), 154 (68), 86 (28), 125 (20), 68 (17)
Hexadecanoic acid	14	256	17.82	55 (100), 73 (85), 60 (82), 129 (25), 87 (23)
cyclo(Val-Phe)	15	246	30.89	91 (100), 55 (58), 127 (42), 85 (37), 99 (30)
1-Hexadecanol	16	252	32.17	55 (100), 69 (60), 83 (53), 97 (45), 111 (27)
cyclo(Pro-Phe)	17	244	33.30	125 (100), 70 (80), 91 (45), 153 (32), 244 (12)
cyclo(Pro-Phe)	18	244	34.70	125 (100), 70 (77), 91 (55), 153 (30), 244 (15)

*M.W. Molecular Weight; ** Rt. Retention time; unfortunately by GC-MS at 70 eV, leucine and isoleucine were indistinguishable

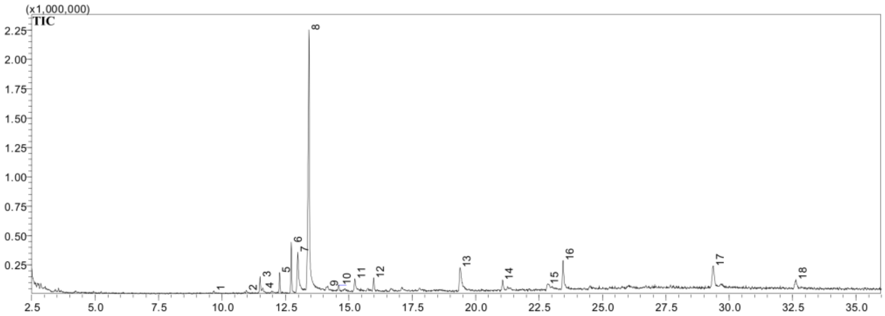

Figure S1. Total ion chromatogram (TIC-GC) of the hexane extract from *X. fastidiosa* 9a5c culture pellet residues grown in PW medium.

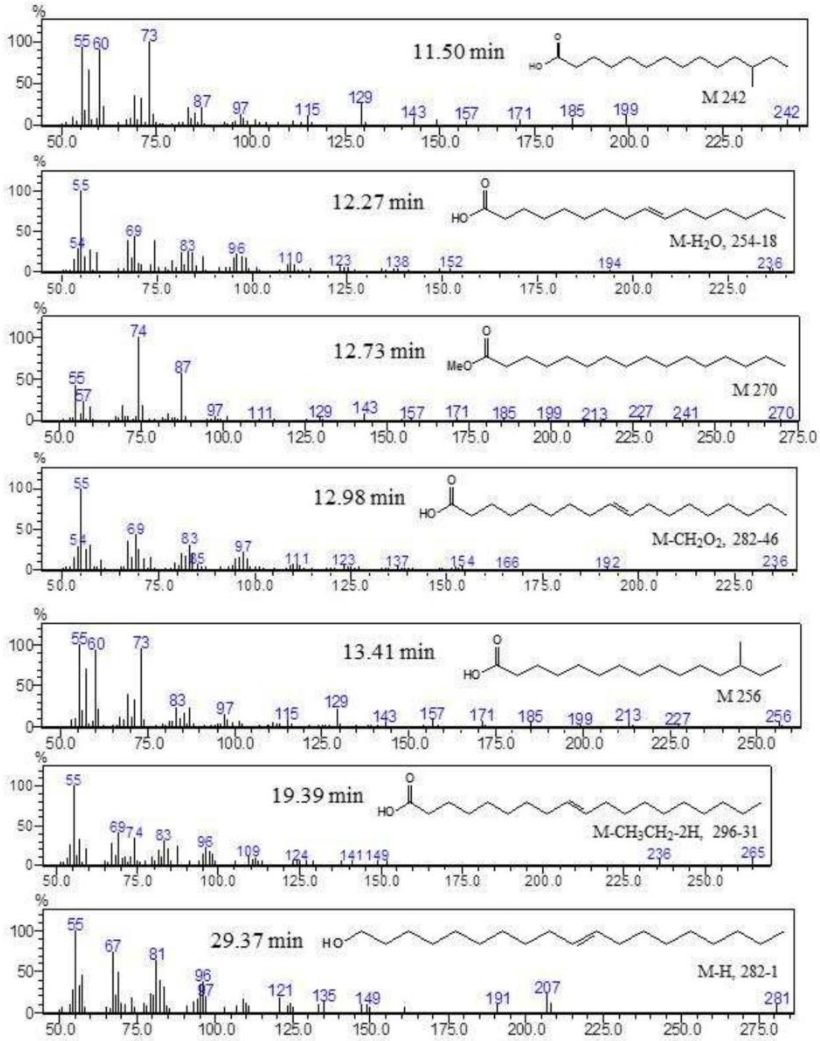

Figure S2. The mass spectra of fatty acids obtained of culture pellet residues from *X. fastidiosa* 9a5c grown in PW medium (GC-MS 70 eV). Peaks were compared with the NIST library.

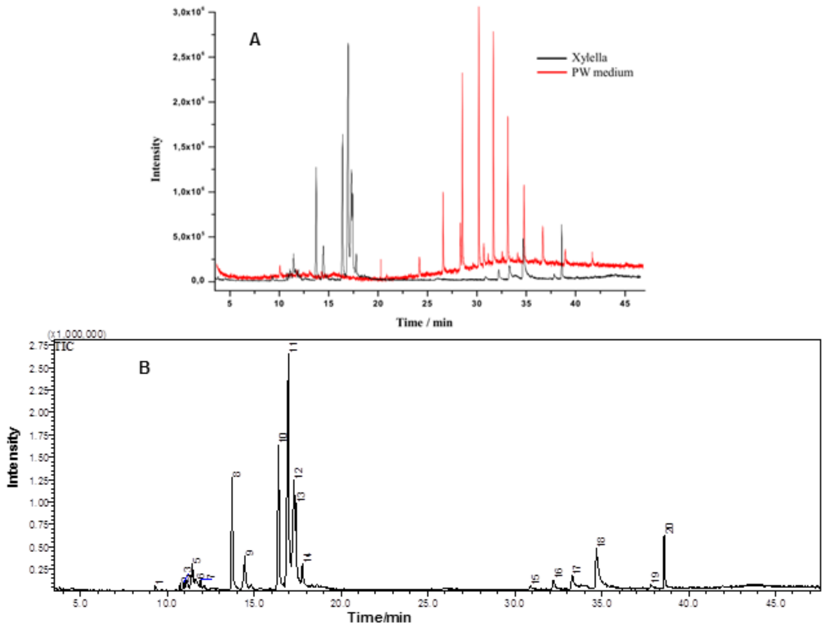

Figure S3. A: Total ion chromatogram (TIC, GC-MS) of the hexane extract from *X. fastidiosa* 9a5c culture supernatant (black) grown in PW medium, and of negative control PW medium (red). B: The total ion chromatogram of diketopiperazines was amplified, and the numbers correspond to the diketopiperazines cited in Table S1.

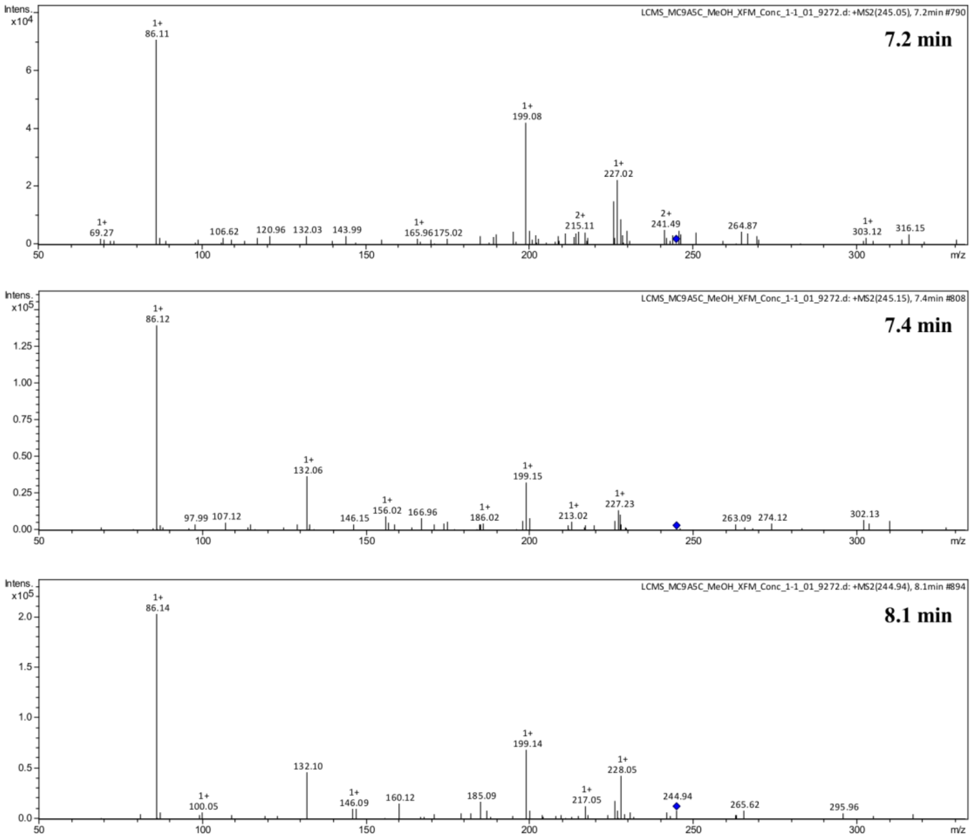

Figure S4. The mass spectra of dipeptides (*m/z* 245) obtained of culture pellet residues from *X. fastidiosa* 9a5c grown in XFM medium (UFLC-ESI-IT, in positive ion mode).

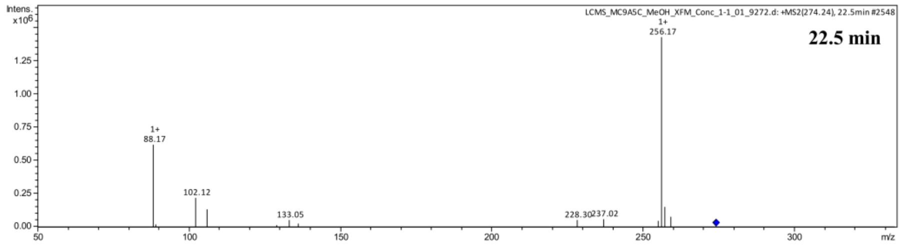

Figure S5. The mass spectra of dipeptides (*m/z* 274) obtained of culture pellet residues from *X. fastidiosa* 9a5c grown in XFM medium (UFLC-ESI-IT, in positive ion mode).

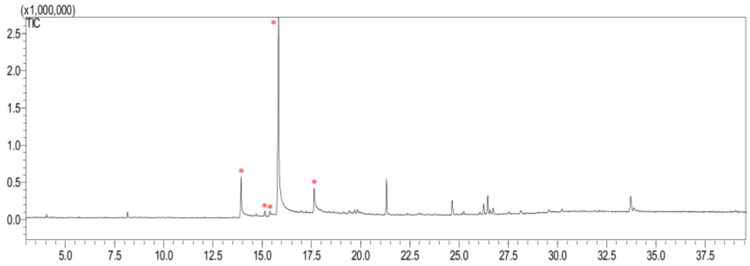

Figure S6. Total ion chromatogram (TIC-GC) of the methanol extract from *X. fastidiosa* 9a5c culture pellet residues grown in XFM medium (GC-MS 70 eV).

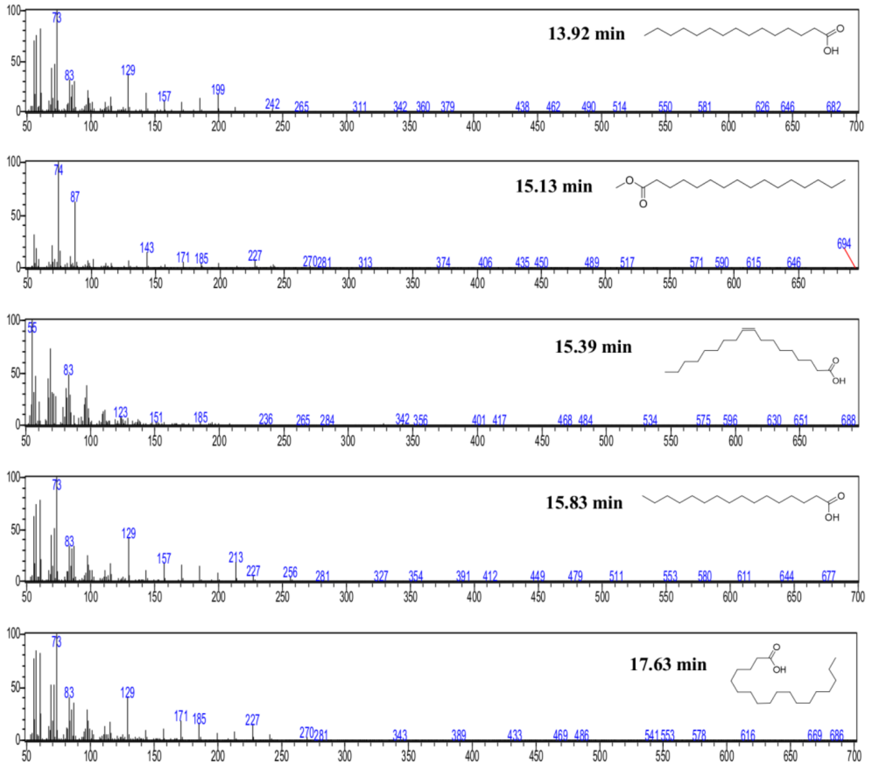

Figure S7. The mass spectra of fatty acids obtained of culture pellet residues from *X. fastidiosa* 9a5c grown in XFM medium (GC-MS 70 eV). Peaks were compared with the NIST library, NIST/EPA/NIH Mass spectral Library (NIST 11): 13.92, similarity 95%, 15.13, 94%; 15.39, 92%; 15.83, 94%; 17.63, 92%.

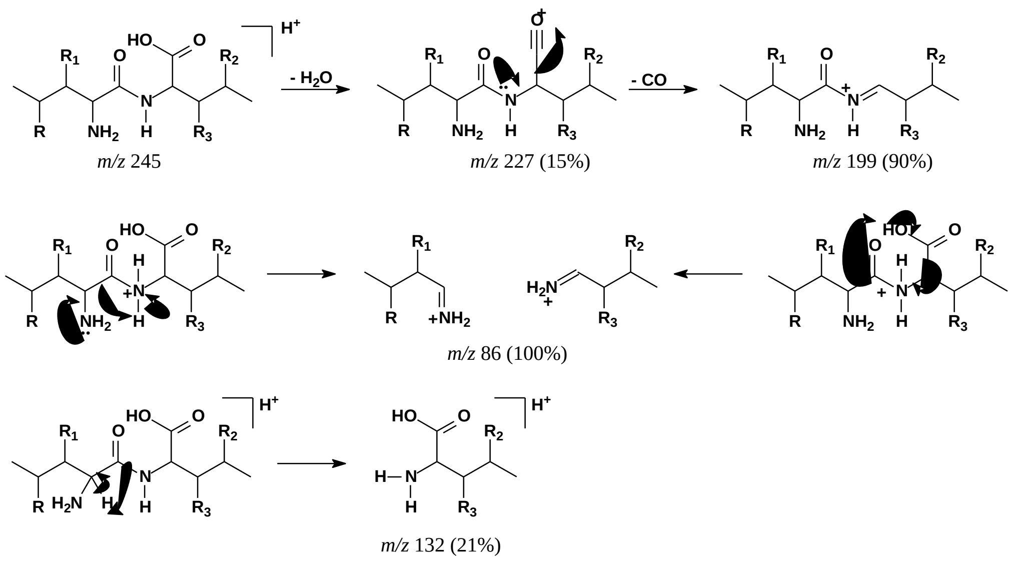

Scheme S1. Fragmentation pathways of dipeptide isoleucyl (R = H, R_1 = Me)-leucine (R_2 = Me, R_3 = H), or leucyl (R = Me, R_1 = H)-isoleucine(R_3 = Me, R_2 = H), or isoleucyl (R = H, R_1 = Me)-isoleucine (R_2 = H, R_3 = Me), or leucyl (R = Me, R_1 = H)-leucine (R_2 = Me, R_3 = H) determined by (+)-ESI-MS/MS.

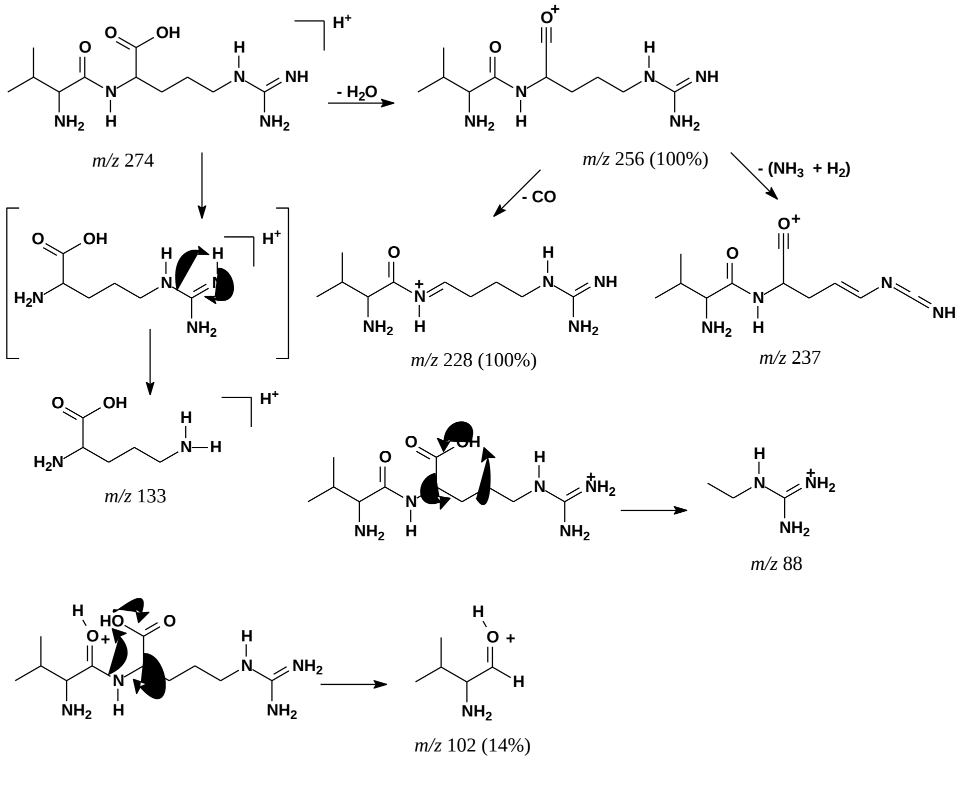

Scheme S2. Fragmentation pathways of dipeptide valyl-arginine determined by (+)-ESI-MS/MS.
